# Effects of Several Bile Acids on the Production of Virulence Factors by *Pseudomonas aeruginosa*

**DOI:** 10.3390/life14121676

**Published:** 2024-12-18

**Authors:** Noureddine Lomri, Christian Hulen

**Affiliations:** Bacterial Communication and Antimicrobial Strategies Research Unit, University of Rouen Normandy, IUT, 55 Rue Saint Germain, 27000 Evreux, France; hulen.marie@orange.fr

**Keywords:** *Pseudomonas aeruginosa*, bile acids exposure, virulence factors production

## Abstract

The presence of bile acids in the cystic fibrosis patient’s lungs contributes to an increase in the inflammatory response, in the dominance of pathogens, as well as in the decline in lung function, increasing morbidity. The aim of this study is to determine the effects of exposure of *Pseudomonas aeruginosa* to primary and secondary bile acids on the production of several virulence factors which are involved in its pathogenic power. The presence of bile acids in the bacterial culture medium had no effect on growth up to a concentration of 1 mM. However, a slight decrease in the adhesion index as well as a reduction in the virulence of the bacteria on the HT29 cell line could be observed. In this model, exposure of *P. aeruginosa* to bile acids showed a significant decrease in the production of LasB and AprA proteases due to the reduction in the expression of their genes. A decrease in pyocyanin production was also observed in relation to the effects of bile acids on the quorum sensing regulators. In order to have an effect on gene expression, it is necessary for bile acids to enter the bacteria. *P. aeruginosa* harbors two potential homologs of the eukaryotic genes encoding the bile acid transporters NTCP1 and NTCP2 that are expressed in hepatocytes and enterocytes, respectively. By carrying out a comparative BLAST-P between the amino acid sequences of the PAO1 proteins and those of NTCP1 and NTCP2, we identified the products of the PA1650 and PA3264 genes as the unique homologs of the two eukaryotic genes. Exposure of the mutant in the PA1650 gene to chenodeoxycholic acid (CDCA) and lithocholic acid (LCA) showed a less significant effect on pyocyanin production than with the isogenic PAO1 strain. Also, no effect of CDCA on the PA3264 gene mutant was observed. This result indicated that CDCA should enter the bacteria by the transporter produced by this gene. The entry of LCA into bacteria seemed more complex and rather responded to a multifactorial system involving the product of the PA1650 gene but also the products of other genes encoding potential transporters.

## 1. Introduction

Chronic respiratory diseases such as asthma, cystic fibrosis, or chronic pulmonary obstruction have emerged in global populations and are among the major causes of mortality [1]. The lungs of healthy people as well as those of sick people have an implanted bacterial community specific to each individual [2]. In the context of a pulmonary infection, bacterial diversity is reduced in favor of a dominant pathogen, as in the case of patients with cystic fibrosis [3,4]. In adults with cystic fibrosis, *Pseudomonas aeruginosa* is the major pathogen, accounting for 70% of the microbiota [5,6,7]. However, the various current treatments have the effect of reducing the predominance of *P. aeruginosa* and increasing biodiversity [8]. Studies carried out in different countries with a large cohort of CF patients have shown the presence of the major pathogen *P. aeruginosa* but also of *Burkholderia cepacia*, methicillin-resistant and -sensitive *Staphylococcus aureus*, *Stenotrophomonas maltophilia*, and *Hemophillus influenzae* [9,10].

In CF patients, the lack of lung clearance contributes to the maintenance of this diversity and persistent inflammation [11]. Pulmonary exacerbation in CF patients is also associated with alterations of several signal molecules including bile acids [12]. The presence of bile acids in the respiratory tract of more than 80% of CF patients is the result of gastro-esophageal reflux and tends to increase co-morbidity with *P. aeruginosa*. The presence of bile acids is also associated with increased hypoxia and inflammation with increased FXR-dependent IL-6 production (farnesoid X receptor) [13]. Canalicular bile, produced by polarized hepatocytes that have transporters in their plasma membrane, is a complex mixture that includes 67% bile acids (BAs), 22% phospholipids (PL), 5% cholesterol (Chol), 5% protein, and bile pigments (BPs) like bilirubin. Its flow rate is approximately 750 mL/day, and the PH is between 7.6 and 8.6 [14].

**Figure 1:**
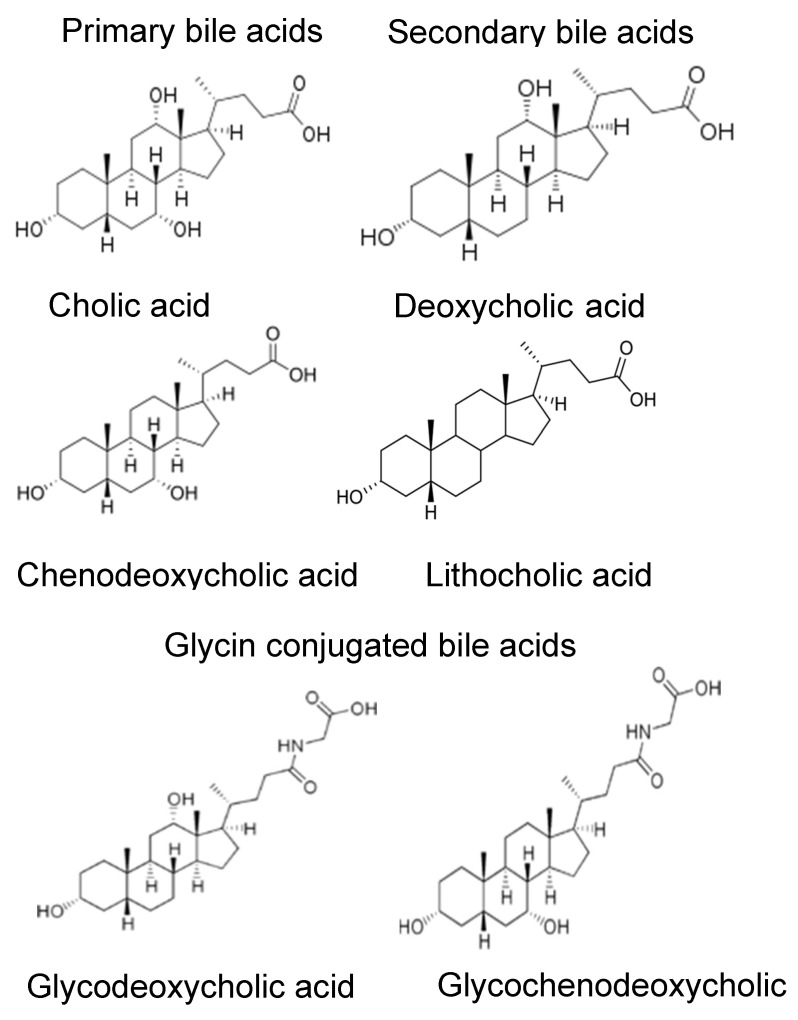
Bile acids used in this study.

Bile acids (BAs) are hydrophobic molecules with detergent properties and are involved in the intestinal absorption of lipids during food digestion. The primary BAs, cholic acid (CA) and chenodeoxycholic acid (CDCA), are synthesized in hepatocytes and released through the bile duct into the intestine upon arrival of the bolus. They are combined with glycine or taurine as well as sodium and potassium ions to form bile salts. A portion of the primary BAs are transformed in the ileum into secondary BAs, deoxycholic acid (DCA), and lithocholic acid (LCA) (Figure 1) by commensal bacteria including those of the genus *Clostridium* [15,16]. About 90% of primary conjugated BAs are then absorbed by the enterocytes of the ileum and colon to be returned to the liver via the portal vein without accumulation in the case of a controlled homeostasis [17]. The dysfunction of this system with the accumulation or absence of release of BAs can cause or contribute to a large number of hepatic and gastrointestinal pathologies such as hepatic lithiasis and ulcerative colitis. These pathologies are currently observed in CF patients, Crohn’s disease, and certain cancers of the colon [18]. Hepatobiliary complications are the third cause of pejorative diagnosis for CF patients [19].

Furthermore, bile acids cause notable effects on the behavior of the intestinal bacterial flora. Indeed, BAs induce a stress gene response in *Escherichia coli*. The micF, osmY, dinD, and AcrAB genes are significantly activated by low concentrations of BAs [20,21]. Another in vitro study carried out on *P. aeruginosa* showed that exposure of bacteria to sub-inhibitory concentrations of bovine bile reduces mobility, promotes bio-film formation, exacerbates quorum sensing (QS), and stimulates the type VI secretion system [22].

Among the many virulence factors secreted by *P. aeruginosa*, we were interested in the effects of bile acid exposure on the production of pyocyanin and proteases. Pyocyanin is an important pigment resulting from the secondary metabolism of bacteria [23]. It contributes to the persistence of infection due to *P. aeruginosa.* This pigment interferes with ion transporters, the frequency of ciliary beats, and the secretion of mucus in aerial epithelial cells [24]. Pyocyanin synthesis is regulated by the quorum sensing elements LasR-LasI, RhlR-RhlI, and PQs, as well as by Vfr [25]. Exposure of *P. aeruginosa* to bile acids induces repression of the *phz* operon, inducing a reduction in pyocyanin production [13].

Three types of proteases secreted by *P. aeruginosa* have been identified: elastase (LasB), anti-staphylococcal protease (LasA), and alkaline protease (AprA). These proteases are associated with the virulence of *P. aeruginosa* by increasing its capacity to destroy matrix structures and epithelial cells to invade deeper tissues. The elastase LasB is considered as a major factor in its virulence. Originally purified and described in 1965 [26], elastase is capable of degrading several biological molecules including elastin, laminin, fibrin, fibronectin, and collagen in human hosts [27,28]. It has also been demonstrated that elastase is involved in the destruction of epithelial cell junctions and signal transduction, disrupting tissue organization [29]. It can also cleave immunoglobulins such as IgG and IgA, complement components, interferon γ, and interleukins, thus inducing a major loss in the host’s immune defense system which can be the cause of autoimmune pathologies [30,31]. Elastase is encoded by the *las*B gene (PA3724 gene in PAO1). The production of active elastase involves three steps: first, the synthesis of prepro-elastase occurs in the bacterial cytoplasm. The latter will be cleaved to form pro-elastase during transport across the cytoplasmic membrane. Elastase becomes active when it is secreted into the extracellular environment. Secretion occurs via the type II secretion system [32]. Expression of the *las*B gene is under the positive control of QS, both by LasR-LasI and RhlR-RhlI [33,34].

After the discovery of the *las*B gene, another gene encoding a protease was discovered: the gene *las*A (PA1871 in PAO1). The protein produced has anti-staphylococcal activity [35,36,37,38,39]. Like the *las*B gene, *las*A transcription is also controlled by the QS *las*R gene [40]. The third protease, alkaline protease, is encoded by the *apr*A gene (PA1249 in PAO1). It has the capacity to suppress host immunity by inactivating the main host defense proteins such as interferon-γ, complement components, and cytokines [41]. It can also inhibit neutrophil function, altering tissues such as LasB and LasA. Gene transcription is also under the positive control of QS, via LasR-LasI [42,43]. We therefore tried in this in vitro study to measure the effects of exposure of *P. aeruginosa* to certain bile acids on the global virulence of the bacterium onto HT29 cells, and on the production of two virulence factors: proteases and pyocyanin.

## 2. Materials and Methods

### 2.1. Bacterial Strains and Culture Medium

Three bacterial strains from collection were used: *Pseudomonas aeruginosa* PAO1 Washington and 2 mutants of *P. aeruginosa* PAO1W in the PA 3264 (PW6477) and PA 1650 (PW3935) genes which would encode Na^+^-dependent transporters as potential bile acid transporters. The mutants were obtained by insertion of the ISlacZ/hah transposon carrying the lacZ reporter gene and a tetracycline resistance gene.

Bacteria were grown in Luria–Bertani (LB) liquid medium (per liter 10 g of Bacto-tryptone, 5 g of yeast extract, 10 g of NaCl) with shaking at 37 °C.

The bacteria are regularly maintained in LB agar medium and stored at −80 °C in LB medium containing 20% glycerol.

### 2.2. Cell Line

Human HT29 cells (ATCC: HTB-38) from colon adenocarcinoma were cultivated in DMEM medium (Dulbecco’s Modified Eagle’s Medium) supplemented with a final 10% fetal bovine serum (FCS), with 2 mM glutamine, 50 μg/mL of streptomycin, and 50 units/mL of penicillin, at 37 °C in the presence of 5% CO_2_.

### 2.3. Chemical Products

All chemicals used in this study were of research grade. Bile acids, deoxycholic acid (DCA), chenodeoxycholic acid (CDCA), lithocholic acid (LCA), glycodeoxycholic acid (GDCA), and glycochenodeoxycholic acid (GCDCA) were supplied by Sigma-Aldrich (St. Quentin Fallavier, France). The stock solutions were prepared at 200 mM in dimethyl sulfoxide (DMSO) for DCA and CDCA, at 200 mM in 50% ethanol/50% water for GDCA and GCDCA, at 100 mM in 75% ethanol/25% water for LCA, and stored at 4 °C.

### 2.4. Effects of Bile Acids on P. aeruginosa PAO1 Growth

Bacteria were recovered after 24 h of growth on an LB agar dish by scraping with a sterile loop and suspended in 500 µL of LB. The bacterial concentration was estimated from measuring the absorbance at 600 nm, and a suspension of 2 × 10^6^ bacteria per mL was made in LB medium. Bile acid solutions at concentrations varying from 0.2 µM to 20 mM were prepared in sterile LB medium except for LCA for which the higher concentration of the sodium salt could not be achieved.

At t_0_ 50 µL of the bacterial suspension (final quantity 1 × 10^5^ bacteria per well) and 50 µL of bile acid solution were added to the wells of a 96-well plate so as to vary the final bile acid concentration from 10 nM to 10 mM. For each bile acid the experiment was carried out on 4 lines of the plate in 3 independent experiments. The absorbance at 595 nm is measured in a µQuant plate reader (Bio-Tek Instruments, Inc., Winooski, VT, USA) and the plates were incubated in a wet chamber at 37 °C. After 24 h of incubation, the absorbance at 595 nm was measured in order to estimate bacterial growth.

The average of the absorbance at 24 h in the wells with bile acid was calculated and compared to the average of the absorbance values in the absence of bile acid. The relative growth values with their standard deviations were then plotted as a function of the bile acid concentration in the wells.

### 2.5. Study of the Virulence of P. aeruginosa PAO1 Treated and Untreated with Bile Acids on HT29 Cells

The bacterial strain PAO1 was grown in 25 mL of LB in a 250 mL Erlenmeyer flask at 37 °C with shaking for 24 h in the absence or presence of bile acids CDCA, GCDCA, DCA, GDCA, and LCA at a final concentration of 1 mM. The supernatant and the pellet were collected after centrifugation of the culture at 10,000× *g* for 5 min. The bacterial pellets were suspended in sterile water, and then a suspension at 5 × 10^8^ bacteria/mL was made in 2 mL of unsupplemented DMEM medium. HT29 cells were recovered by trypsinization of the cell layer of a T75 dish at 90% confluence and diluted to 80 mL in supplemented DMEM medium.

#### 2.5.1. Measurement of the Adhesion Index

HT29 cells were grown in supplemented DMEM medium at 70% confluence in a 24-well plate on a coverslip at 37 °C with 5% CO_2_. Then, cells were washed once in unsupplemented DMEM medium and placed in the presence of 5 × 10^8^ PAO1 in 500 µL of DMEM. After 3 h of incubation at 37 °C in a microbiological oven, the bacteria are eliminated; the cells were gently washed 4 times with preheated PBS and then fixed in 500 µL of methanol for 10 min. The cells were then stained for 30 min at room temperature with 10% (*v*/*v*) Geimsa dye. The cells were washed 3 to 4 times with distilled water and the coverslips were sticked to a glass slide for subsequent analysis by optical microscopy. The count of adherent bacteria was carried out on 2 × 100 HT29 cells on each coverslip. The index measurement was carried out twice in triplicate: 3 lines under the same conditions and 6 columns with the different bile acid treatments: untreated, CDCA, GCDCA, DCA, GDCA, and LCA.

#### 2.5.2. Measurement of the Toxicity of PAO1 Culture Supernatants

In a 24-well plate containing confluent HT29 cells in DMEM medium, increasing volumes (100 µL, 10 µL, 1 µL, 0.1 µL (1 µL of a dilution 10)) of supernatants concentrated twice on a PEG bed, from PAO1 culture treated or not with bile acids, were added in each well (one column for a given sample). After 48 h of incubation at 37 °C, the culture medium was removed; the cells were washed twice with DMEM medium and then fixed in methanol for 10 min at room temperature. The cells were then stained with 500 µL of 0.1% crystal violet for 30 min at 37 °C. The dye was then removed, the cells washed 4 times with PBS, and then the dye fixed in the cells was dissolved in 1 mL of 1% SDS. The absorbance at 595 nm was measured in a µQuant plate reader (Bio-Tek Instruments, Inc.). The result of the virulence of the supernatant is estimated by the ratio of absorbance between treated and untreated bacteria.

#### 2.5.3. Measurement of the Virulence of Bacteria Treated with Bile Acids on HT29 Cells

HT29 cells were grown in 24-well plates until confluence. After elimination of the culture medium and washing the cells once with unsupplemented DMEM medium, cells were exposed for 4 h at 37 °C to the presence of 5 × 10^8^ PAO1 in 1 mL of unsupplemented DMEM medium. After incubation, the bacteria were eliminated, and the cells washed 4 times with PBS, before being fixed with methanol. The cells were then stained for 30 min with 0.1% crystal violet. After removing the dye and washing the cells, they were lysed in 1 mL of 1% SDS. The absorbance at 595 nm was measured in a plate reader and the virulence of the bacteria estimated by the ratio of absorbance between treated and untreated bacteria. Loss of virulence is expressed in percent using untreated bacteria as maximum mortality of HT29 cells.

### 2.6. Effects of Bile Acids on the Production of Virulence Factors

#### 2.6.1. Recovery of Supernatants and Protein Assay

*P. aeruginosa* PAO1 was grown at 37 °C for 24 h either in 25 mL of LB with shaking in an Erlenmeyer flask or in 6 mL of LB with shaking in a 50 mL Falcon tube. The 2 types of culture at different volumes and in different containers were carried out to obtain 2 different growth conditions in relation to oxygen availability. The air/liquid exchange surface during stirring is 10 times greater in the Erlenmeyer flask than in the Falcon tube and, consequently, the quantity of dissolved oxygen.

After growth, bacteria were centrifuged at 10,000× *g* for 5 min. The bacterial pellet was frozen at −20 °C while awaiting RNA extraction and the supernatant containing the soluble secreted proteins was filtered on through a sterile HAWP Millipore filter in the amount of 0.45 µm. On another hand, to recover the proteins secreted by the bacteria during their growth, two techniques were used.

Proteins present in the supernatant were precipitated by ammonium sulfate. For 20 mL of supernatant, 12 g of ammonium sulfate was added gradually to reach a saturation percentage of 90%. The proteins were then recovered by centrifugation at 12,000× *g* for 15 min. The supernatant was removed and the pellet suspended in 4 mL of 50 mM Tris-HCl buffer, pH 8. On another hand, proteins present in the supernatant were concentrated in dialysis hoses on a bed of polyethylene glycol (PEG). An amount of 20 mL of supernatant was placed in a dialysis tube (Medicell International Ltd., London, UK) with a porosity of 12,000 to 14,000 Daltons. The dialysis tube was placed on a PEG bed and covered with PEG. The water outlet was carried out at room temperature for 4 to 6 h. The concentrate was recovered and the tube washed with 50 mM Tris-HCl buffer, pH 8, for a final volume of 4 mL.

Protein concentration was measured with the Bradford reagent (BioRad protein assay). Samples of different volumes were placed in the wells of a 96-well plate and distilled water was added to each well for a final volume of 160 µL. Protein staining is carried out by adding 40 μL of Bradford reagent. In the same plate, a standard range ranging from 0.5 to 5 µg of bovine serum albumin (BSA) is produced. The absorbance is read at 595 nm in the plate reader. The protein concentration of the samples is calculated from the slope of the standard line.

#### 2.6.2. Measurement of Protease Activities by Hydrolysis of Azocasein

Protease activity was measured by hydrolysis of azocasein. Different volumes of samples were incubated for 10 min at 37 °C in a total volume of 500 µL containing 300 µL of 50 mM Tris-HCl pH8 buffer and a final concentration of 0.6% azocasein. After incubation, 100 μL of 50% trichloroacetic acid (TCA) was added and the tubes were placed on ice for 5 min. After centrifugation of the tubes for 10 min at 13,000× *g*, the supernatant was recovered and 200 µL of 1N NaOH was added to neutralize the TCA. The absorbance at 440 nm was measured on each tube. The reaction rate and the specific activity were calculated and expressed, respectively, in mg of azocasein hydrolyzed per minute by using OD = εlC with ε% = 36, and in mg of azocasein hydrolyzed per minute and per mg of protein in the samples using.

#### 2.6.3. Measurement of Pyocyanin Production

The demonstration of the production of pyocyanin in the culture medium of *P. aeruginosa* PAO1 treated or not with bile acids was carried out by measuring the absorbance at 690 nm. This value for the treated samples is compared to that of the untreated sample. From the supernatant containing the pyocyanin, the quantification was carried out either directly with the spectrophotometer with the measurement of the absorbance or after the extraction of pyocyanin with chloroform. No difference in the absorbance values was observed revealing a yield of 100% during the extraction of pyocyanin by chloroform. Pyocyanin was quantified by using the following formula: pyocyanin concentration (mg/mL) = absorbance at 690 nm ÷ 16 [44].

### 2.7. Extraction of RNA

RNA extraction was carried out according to the protocol given by the Extract-All manufacturer. One hundred milligrams of bacteria treated or not with bile acids were suspended in 200 µL of sterile water. An amount of 1 mL of the Extract-All solution was added and the tubes were shaken vigorously then left for 5 min at room temperature. An amount of 200 µL of chloroform was then added to each tube, and then they were centrifuged at 12,000× *g* for 15 min and at 4 °C. After centrifugation, the homogenate presents three phases: the lower red phenol–chloroform phase, the whitish colored interphase, and the upper colorless aqueous phase which exclusively contains the RNA. Around 600 µL of this aqueous phase was collected in clean tubes RNases-free to which 500 µL of isopropanol was added to precipitate the RNA. After 10 min at room temperature, the tubes were centrifuged at 12,000× *g* for 10 min at 4 °C and the RNA which had formed a whitish precipitate at the bottom of the tube was washed once with 75% ethanol. After 5 min of centrifugation at 12,000× *g*, the RNA pellet was dried in the open air and then dissolved in RNase-free water. The Extract-All method used for the extraction of RNA from the bacterial pellet made it possible to obtain a sufficiently large quantity of RNA. Measurement of the 260 nm/280 nm absorbance ratio which gave a value between 1.8 and 1.95 testifies to the purity of the extract.

### 2.8. RT-PCR

After extraction of the RNA, 10 μg of each RNA sample is reverse-transcribed into complementary DNA (cDNA) by M-MLV reverse transcriptase using a set of random primers (random primers from Promega) and according to the protocol established by the supplier. Briefly, to 10 µg of RNA in the solution are added 2 µL of random primers and 2 µL of 2.5 mM dNTP, all supplemented to 20 µL with H_2_O RNase-free and incubated for 5 min at 65 °C, then placed on ice. Then, 1 µL of RNase inhibitor, 8 µL of 5X buffer, and 2 µL of M-MLV RT were added to the first mixture; then, the volume was made up to a final 40 µL with H_2_O RNase-free. The reaction mixture was incubated for 1 h at 37 °C, then 10 min at 70 °C. The tubes are stored in the freezer at −20 °C.

The PCR reaction was then carried out with pairs of primers specific for the elastase (*las*B), alkaline protease (*apr*A), and pseudaminidase (*nan*A) genes, with *rDNA* 16S as the housekeeping gene (see table below).


**DNA Synthesis: Primers Used in This Study**

**Gene**

**Amplicon Size**

**Fw Primer Sequence**

**Rev Primer Sequence**
*las*B261 bpGCCGCAAGACCGAGAATGAGTCGTTGACGATCAGCGGAC*apr*A488 bpTACGGCTTCAACTCCAACACTCGACGTATTGCAGCACCA*nan*2893 bpCCTTGCAAACTCAACGACCAAGGCGGGAGGACAGGATTTT*rDNA*16S202 bpAAGCAACGCGAAGAACCTTAAAGCAACGCGAAGAACCTTA

A volume of 2 µL of complementary DNA was added to the final 50 µL PCR solution (5 µL 5X buffer, 5 µL of 10 mM MgCl_2_, 1 µL of a tenfold dilution of each primer, 2 µL of a dNTP solution at 2.5 mM, 2 µL of Taq DNA Polymerase and H_2_O DNase RNase free qsp 50 µL). Amplicon synthesis was performed for 15, 20, or 25 cycles. After denaturation of the cDNA at 94 °C for 5 min, the cycles alternated: 94 °C for 30 s, 60 °C for 30 s, 72 °C for 30 s, and after the desired number of cycles elongation for 7 min at 72 °C. The amplification products were separated by electrophoresis on a 1.2% agarose gel containing ethidium bromide (100 V migration for 30 min). The intensity of the bands for their comparison was analyzed on the photos of the gels using KODAK 1D Image Analysis Software version 3.5.4. For each amplification, the intensity of the band was compared to that obtained after amplification of the 16S rDNA and then compared with the bands resulting from the amplifications from the cDNAs of the treated and untreated bacteria.

### 2.9. Mathematical Analysis

Comparisons of significance of variation of virulence factors production in the presence of BAs were obtained by the Fisher–Snedecor variance analysis test. The *p*-values were calculated from the numerical values for each pair of bile acid-treated bacteria and untreated bacteria. The *p*-values associated with each percentage of inhibition are given in the tables.

## 3. Results and Discussion

### 3.1. Effects of Bile Acids on the Growth of P. aeruginosa PAO1

**Figure 2:**
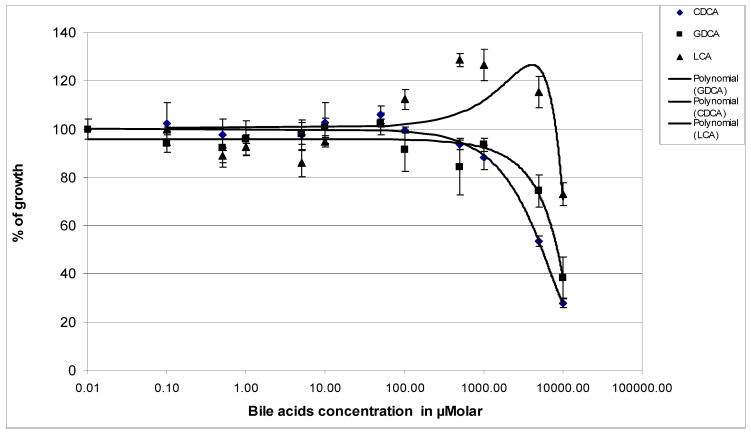
Effects of 24 h exposure to increasing CDCA, GDCA, and LCA concentrations on the growth of *P. aeruginosa* PAO1. Bacteria were grown for 24 h in a 96-well plate containing increasing concentrations of bile acids at 37 °C in a wet chamber. The absorbance at 595 nm is measured in a µQuant plate reader at t_0_ and at t_24_. The average of the absorbance at 24 h in the wells with bile acid was calculated and compared to the average of the absorbance values in the absence of bile acid. The relative growth value with its standard deviation was then plotted as a function of the bile acid concentration in the wells. Results are the mean values of three independent experiments.

In CF patients, *P. aeruginosa* is part of the infection flora and is distributed throughout the body, including the intestines as passing flora and the lungs as resident bacteria. In both cases it may be exposed to bile acids. We first investigated whether exposure of *P. aeruginosa* PAO1 to bile acids had an influence on growth. The bacteria were grown for 24 h in a 96-well plate containing increasing concentrations of BAs as described in Materials and Methods. The results of exposure of PAO1 to chenodeoxycholic acid (CDCA), a primary BA, and glycodeoxycholic acid (GDCA), a secondary BA, for 24 h in LB medium are presented in Figure 2. The presence of CDCA and GDCA did not prevent the growth of the bacteria up to a concentration of 1 mM bile acid. Beyond this concentration we observed a significant reduction in biomass production. BAs being very hydrophobic organic molecules derived from cholesterol, they were dissolved in DMSO or ethanol. It might be possible that the lack of growth in the 5 and the 10 mM BAs wells should be due to the high concentration of either DMSO or ethanol as chaotropic agents and the high concentration in bile acid as detergent. On the other hand, the effect of LCA on the growth of PAO1 seemed different. Indeed, from a concentration of 0.1 mM of LCA growth was stimulated by the presence of bile acid by approximately 30%. At a concentration of 5 mM of LCA we observed a decrease in growth as that observed with other BAs. For the rest of the experiments, we have chosen to treat the bacteria with 1 mM bile acids.

### 3.2. Effects of Exposure of P. aeruginosa PAO1 to 1 mM Bile Acids on the Virulence of the Bacteria on HT29 Cell Line

#### 3.2.1. Adhesion Index Measurement

PAO1 was grown in 25 mL of LB at 37 °C with shaking for 24 h in the absence or presence of bile acids CDCA, GCDCA, DCA, GDCA, and LCA at a final concentration of 1 mM. Bacteria were collected by centrifugation and suspended in 2 mL of unsupplemented DMEM medium. The adhesion index was measured after 3 h of incubation of PAO1 with 70% confluent HT29 cells at 37 °C. The results are presented in Table 1.

The adhesion index of untreated *P. aeruginosa* to BAs was in the order of magnitude of the indices observed with cells which present a high level of sialic acid on their extracellular matrix proteins, thus masking the specific binding sites of the bacteria. The adhesion index observed in Table 1 did not show an important difference between bacteria exposed to bile acids and bacteria not exposed. However, we could still note a 30% decrease in the adhesion capacity of PAO1 after treatment with DCA, GDCA, and GCDCA, and 15% after exposure to LCA. It seemed that exposure of *P. aeruginosa* to 1 mM bile acids during its growth in a liquid medium should modify the surface properties of the bacteria, resulting in a slight reduction in its adhesion capabilities.

#### 3.2.2. Virulence of the *P. aeruginosa* Growth Supernatants on HT29 Cells

Supernatants used in these experiments were from a PAO1 culture grown for 24 h at 37 °C with stirring in 25 mL LB medium containing or not containing 1 mM bile acids. Supernatants were concentrated twice in dialysis hoses on a bed of PEG. Increasing volumes of supernatants were added to confluent HT29 cells and incubated for 48 h at 37 °C and 5% CO_2_. Cells exposed to bacterial supernatants (exotoxins) died and the presence of proteases promoted their separation from the cell layer. The viability of the cells after exposure or not to bile acids was deduced after crystal violet staining of the cells remaining attached to the dish by measuring the OD at 595 nm. The absorbance at 595 nm of the cells without any contact with the supernatants represents 100% survival.

**Figure 3:**
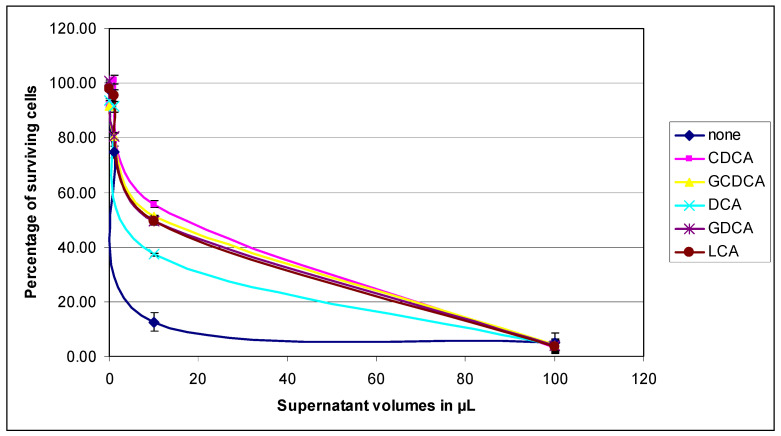
Percentage of HT29 cells surviving after 48 h of exposure to PAO1 culture supernatants treated or not with bile acids. PAO1 was grown for 24 h at 37 °C with shaking in 25 mL LB medium in the presence or absence of 1 mM bile acids. After centrifugation of the bacteria, the supernatants were concentrated twice in a dialysis hose on a PEG bed. Increasing amounts of supernatants from 0.1 to 100 µL were added to confluent HT29 cells and incubated for 48 h at 37 °C in a CO_2_ incubator. Then, the cells were washed gently with DMEM medium and the adherent cells were stained with 0.1% crystal violet. After removal of the dye, the adherent cells were washed then lysed with 1% SDS. The absorbance at 595 nm was measured in a plate reader and the absorbance values of the wells containing the supernatants of the bile acid-treated bacteria compared to those obtained with the supernatants of the untreated bacteria were measured. Mean values with SD reported in the figure were obtained by the average of the values resulting from three independent experiments carried out under the same conditions.

As shown in Figure 3, treatment of bacteria with bile acids decreased their virulence power. For 100 µL of supernatant the percentage of survival between untreated and treated bacteria was not very different; it varies between 5% as the highest for the supernatant obtained in the culture without treatment, and 3.3% as the lowest obtained in bacteria treated with CDCA. All supernatants tested contained enough bacterial toxins to kill more than 95% of cells. On the other hand, for 10 µL of supernatant, the survival percentage of HT29 cells was greater and varied between 55% survival for the CDCA treatment versus 12% survival for the untreated. It appeared that the supernatants of treated bacteria were up to four times less virulent than the supernatant of untreated bacteria. It is possible that overall, the amount of virulence factors in the supernatants of bile acid-treated bacteria was less than in the supernatant of untreated bacteria.

#### 3.2.3. Virulence of the PAO1 Strain on the HT29 Cells

**Figure 4:**
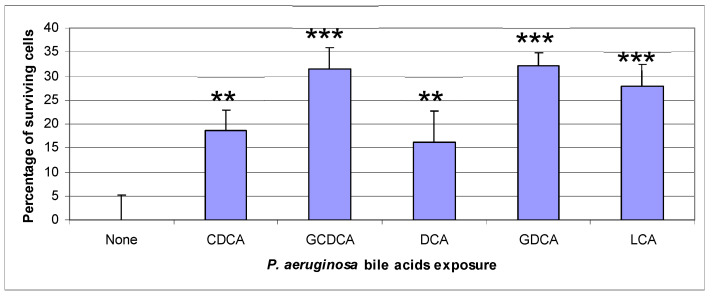
Measurement of *P. aeruginosa* virulence on HT29 cells after treatment of the bacteria with bile acids. PAO1 was grown for 24 h at 37 °C with shaking in LB medium in the presence or absence of 1 mM bile acids. After centrifugation, 5 × 10^8^ bacteria were suspended in 1 mL unsupplemented DMEM and added to confluent HT29 cells in a 24-well plate and incubated for 4 h at 37 °C with 5% CO_2_. Then, the cells were washed gently with unsupplemented DMEM medium and the adherent cells were stained with 0.1% crystal violet. After removal of the dye, the adherent cells were washed, and then lysed with 1% SDS. Absorbance at 595 nm was measured in a plate reader. The mean value with the SD for untreated bacteria was compared to the mean values of treated bacteria to determine the percentage of surviving cells. The *p*-values associated with each percentage of inhibition were obtained by the Fisher–Snedecor variance analysis test (*** *p* < 0.05, ** *p* < 0.1).

Bacteria were grown in 6 mL of LB for 24 h at 37 °C with shaking, under the six conditions tested previously. Bacteria recovered after centrifugation were incubated for 4 h with confluent HT29 cells in a 24-well plate in triplicate. After washing the wells with unsupplemented DMEM, the adherent cells were stained with crystal violet, and then the absorbance at 595 nm was measured. The absorbance value in each well was directly proportional to the number of surviving cells. The results are presented in Figure 4.

The first column of Figure 4 shows the virulence of untreated PAO1 on HT29 cells with maximum mortality and was used as reference (0% of surviving cells). For bacteria treated with BAs we observed a greater survival of HT29 cells associated to a loss of virulence in the same order of magnitude as the decrease in the adhesion index. The cell survival rate can be estimated at 19% with CDCA-treated bacteria, and 31%, 16%, 32%, and 28% with GCDCA-, DCA-, GDCA-, and LCA-treated bacteria, respectively. This loss of killing capacity of *P. aeruginosa* could be explained both by the decrease in its adhesion capacities (Table 1) and by the loss of virulence through a reduction in the production of toxins (Figure 3).

### 3.3. Effects of Bile Acids on the Production of Virulence Factors of P. aeruginosa PAO1

#### 3.3.1. Protease Activities in PAO1 Culture Supernatants

PAO1 was grown for 24 h in LB liquid medium in the presence of BAs at a concentration of 1 mM under different volumes and shaking conditions. The supernatants were recovered and the secreted proteins were concentrated either by ammonium sulfate precipitation or by elimination of liquids on a PEG bed in a dialysis hose. The presence of proteases was detected by measuring the hydrolysis of azocasein.

Table 2 presents the values of the specific activities of the proteases present in the supernatants of a 24 h culture in 25 mL in an Erlenmeyer flask at 37 °C with vigorous stirring in the presence or absence of 1 mM bile acids in the medium. Proteins were concentrated twice in a dialysis hose on a PEG bed. The specific activity for proteases was expressed as mg of hydrolyzed azocasein per minute and per mg of total proteins in the supernatant. In the absence of treatment and under the chosen culture conditions, the specific activity in the supernatant was 0.542 ± 0.082 mg azocasein hydrolized/min/mg of secreted proteins. The presence of BAs in the culture medium at 1 mM during growth caused a more or less significant reduction in the specific protease activity with the maximum loss for LCA (around 33%) and the minimum for DCA (approximately 2.5%). Primary bile acid CDCA and its derivative glycine ester glycochenodeoxycholic had intermediate values (22% and 20%, respectively).

Table 3 presents the specific protease activity values in the supernatants of PAO1 cultures carried out in a volume of 6 mL of LB in 50 mL Falcon tubes, in the absence or presence of 1 mM BAs. Proteins in the supernatants were concentrated five times by ammonium sulfate precipitation.

First of all, we can notice that the specific protease activity in the culture supernatant of untreated PAO1 was greater than that found in the previous experiment. The observed multiplicative factor of 2.5 corresponds to the difference between the concentration factors of the supernatants, two times for growth in the Erlenmeyer flask, and five times for growth in the Falcon tube.

However, the loss of activity observed with bacteria exposed to bile acids was much greater under these low-volume growth conditions (6 mL in Falcon tubes). The greatest losses were observed in the supernatants of the cultures in the presence of GCDCA, GDCA, and LCA. DCA, which induced a very low loss of activity in Erlenmeyer cultures, causes here 44% loss of activity. The difference might be explained by the fact that the availability of oxygen, which represents an important element for the growth of this aerobic bacteria and the triggering of the quorum sensing (QS) effect, is less important during growth in the Falcon tube than in the Erlenmeyer flask.

To try to understand the origin of the effects observed on protease activity and to know if the production of proteases is really reduced, we analyzed, after extraction of total RNA, the expression of two genes producing proteases by RT-PCR.

#### 3.3.2. Study of the Transcription of Elastase, Alkaline Protease, and Pseudaminidase Genes by RT-PCR

In order to confirm the results obtained previously, which showed a decrease in protease activity in PAO1 culture supernatants in the presence of BAs, we analyzed by RT-PCR the expression level of *las*B and *apr*A genes. We had also amplified an internal segment of the *nan*A gene encoding the pseudaminidase, another virulence factor involved in the adhesion of bacteria to cells.

Bacteria were grown in 6 mL LB in a Falcon tube in the presence or absence of BAs, and after centrifugation they were lysed with Extract All to recover the total RNA following the protocol of the manufacturer. Then, the complementary DNAs were obtained by reverse transcription using random primers. The presence of specific cDNAs encoding elastase, alkaline protease, and pseudaminidase was demonstrated by PCR using primers constructed within these genes (see Materials and Methods). To clearly identify a possible variation in the expression level of each gene, several series of increasing cycles of amplicon synthesis were carried out (15 cycles, 20 cycles, 25 cycles). The PCR products were analyzed by migration on 1.2% agarose gel.

**Figure 5:**
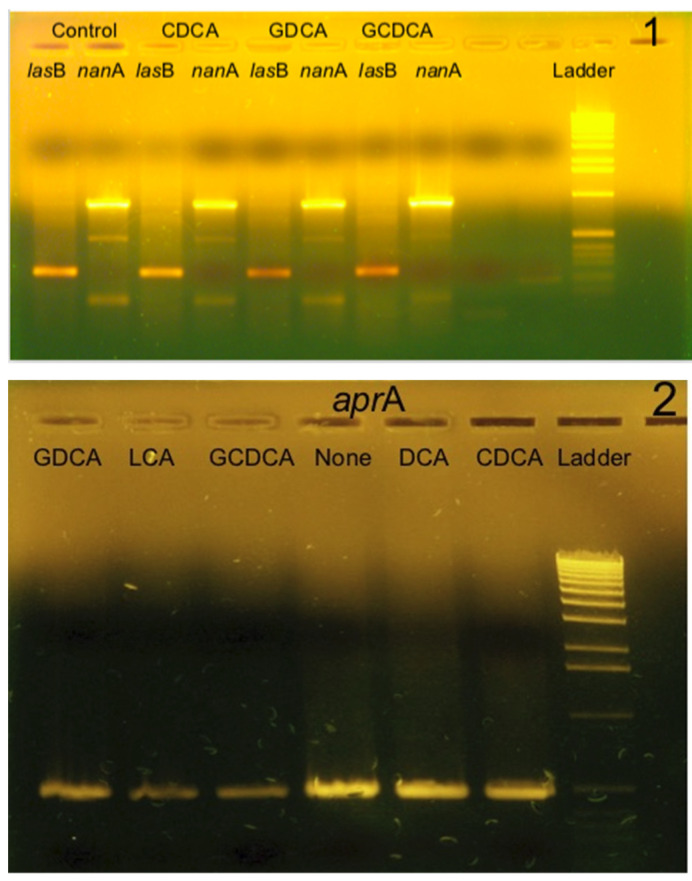
Visualization on 1.2% agarose gel of the RT-PCR products (20 cycles) of the genes encoding elastase (*las*B: amplicon 261 bp), pseudaminidase (*nan*A: amplicon 893 bp) gel 1, and alkaline protease (*apr*A: amplicon 488 bp) gel 2, following the treatments of bacteria with 1 mM bile acids.

Figure 5 shows an example of the RT-PCR results. The expression levels of the *las*B and *apr*A genes, gel 1 and gel 2, respectively, were different depending on the type of BA present during the growth of the bacteria. On the other hand, the expression of the *nan*A gene did not seem to be affected by the presence of BA. Next, the intensity of each amplicon on each gel was analyzed using a photo imager (KODAK 1D Image Analysis Software version 3.5.4). The intensity of the band for each amplification was reported to the intensity of the band obtained by amplification of the 16S rDNA in the same bacterium, and then the intensities of the amplicons were compared with each other. The percentages of inhibition of the expression of the three genes, *las*B, *apr*A, and *nan*A, are presented in Table 4.

We can observe in Table 4 that the expression rate of the two genes *las*B and *apr*A was reduced when bacteria were grown in the presence of BAs with a major effect for exposure to LCA and GDCA. The decrease in the expression level of the genes encoding the two proteases LasB and AprA resulted in a reduction in the quantity of these enzymes in the culture supernatants. The sums of the percentages of inhibition of the expression levels of the two genes for each exposure to bile acids are of the same order of magnitude as the rate of inhibition of protease activity in each of the supernatants (Table 2 and Table 3).

On the other hand, the inhibition values observed for the expression of the *nan*A gene were not significant and, as shown in Figure 5, the expression of this gene was not inhibited in the bacteria exposed to BAs.

### 3.4. Effect of Bile Acids on the Production of Pyocyanin by PAO1

PAO1 was grown for 24 h at 37 °C in LB liquid medium with vigorous shaking in the presence of BAs at a concentration of 1 mM under different volumes, 25 mL in a 250 mL Erlenmeyer and 6 mL in a 50 mL Falcon tube. After growth, the production of pyocyanin was measured by absorbance at 690 nm directly on the supernatant or after extraction with chloroform, as described in Materials and Methods. The pyocyanin concentration is calculated from the following formula: absorbance at 690 nm ÷ 16 = pyocyanin concentration (mg/mL) [44]. The results obtained are presented in Table 5 and Table 6.

The production of pyocyanin was strongly inhibited in treated bacteria. The percentage of inhibition varies between 43% and 73%, being the lowest with CDCA and the highest with LCA. The synthesis of the pyocyanin virulence factor was regulated by the Rhl system of the quorum sensing. The reduction in its production by bacteria grown in the presence of BAs confirms the repression yet observed on *phz*A–E genes, products of which are necessary for pyocyanin production [13].

After growth in the Falcon tube, we observed a general decrease in pyocyanin production accompanied by a decrease in the inhibitory effect of BAs compared to growth in an Erlenmeyer flask, except for LCA, the most hydrophobic bile acid. The air/liquid exchange surface being lower in the Falcon tube than in the Erlenmeyer flask, consequently, the availability of oxygen must be lower without having any impact on biomass production in 24 h. The speed of growth should only be slowed down. We also observed the opposite effect to that observed for the production of proteases. The percentage of inhibition of pyocyanin production by PAO1 in the Falcon tube was lower than that observed with PAO1 grown in the Erlenmeyer flask, except for LCA. However, we were able to observe the opposite effect for the production of proteases where the percentage of inhibition was higher for the PAO1 culture in the Falcon tube.

In these two cases, the availability of oxygen plays an important role in the activation of the effects of bile acids on the expression of the genes concerned in both positive and negative ways. Culturing in the Erlenmeyer flask allows for a greater surface area for exchange between the bacteria and the medium; on the other hand, culturing in the 6 mL reduced the level of available oxygen. It seemed that the regulatory system for the expression of genes involved in the production of proteases and pyocyanin was involved. The expression of the *las*B and *apr*A genes, and the genes responsible for phenazine synthesis, were under the control of quorum sensing regulators LasR/RhlR and, more particularly, with regard to the effect of oxygen, under the control of the major membrane sensor GacS sensitive to oxidative stress and cell density [33,45,46]. The GacS/GacA/Rsm system acts as a global regulator on the production of virulence factors, biofilm formation, or planktonic lifestyle [47,48].

### 3.5. Search for Potential Bile Acids Transporters in P. aeruginosa PAO1

The enterohepatic circulation of BAs is fundamentally composed of two major processes: secretion by the liver and absorption by the intestine. Specific transporters are expressed in hepatocytes of the liver and enterocytes of the intestine. They play an essential role in maintaining bile acid and cholesterol homeostasis.

BAs synthesized in hepatocytes are secreted into the canaliculi connecting two hepatocytes together by the BSEP transporter (Bile Salt Export Pump, gene ABCB11). Phospholipids are transported via MDR2 (gene ABCB4), a multidrug transporter, and cholesterol is transported by different transporters belonging to the ATP-binding cassette protein family (genes ABCG5/ABCG8). Other components needed to form bile use other transporters. Bilirubin and organic anions use the transporter ABCC2 (MRP2), phospholipids MDR3 and phosphatidylserine flippase use ATP8B1 [16,49]. In the intestine, most primary and secondary BAs are reabsorbed by the transporter ASBT (apical sodium-dependent bile acid transporter NTCP2, gene SLC10A2) at the level of enterocytes in the final part of the small intestine and upstream of the colon. They are then released into the circulation via the Ostα/Ostβ bile acid transporters (organic solute transporters) and return to the liver via the portal vein. BAs enter the hepatocytes via the transporters NTCP1 (Na+/taurocholate cotransporting polypeptide, gene SLC10A1) and OATP (solute carrier organic anion transporter), where they will be conjugated again, except for LCA which is sulfoconjugated or oxidized to ursodeoxycholate (UDCA), a tertiary bile acid. The resulting primary and secondary BAs are excreted back into the bile using BSEP [49].

We recovered the amino acid sequences of the proteins NTCP1 (Q14973) and NTCP2 (Q12908), the transporters for BAs in hepatocytes and enterocytes, respectively, and we performed a BLAST-P against proteins of *P. aeruginosa* PAO1 on the website Pseudomonas.com. For the two eukaryotic transporters we obtained the same answer. Two candidates with the best scores were selected. Each of the genes involved encodes a potential Na^+^-dependent transporter. These are the products of the PA1650 and PA3264 genes. The PA1650 gene product has 25.5% identity with NTCP1 and 24.5% identity with NTCP2. The PA3264 gene product has 26% identity with NTCP1 and 28% with NTCP2. Then, we recovered from the PAO1 Washington library two mutants in these genes, PW3935 for the PA1650 gene and PW6476 for the PA3264 gene. Since secondary bile acids are involved in the enterohepatic circulation, we used LCA and its precursor CDCA in the next studies. We therefore tested the production of pyocyanin by these two mutants after growth in the presence of 1 mM CDCA or LCA by comparison with the production in the isogenic strain. The results are presented in Table 7.

The control carried out with the isogenic wild strain PAO1 Washington showed a pyocyanin production rate greater than values recorded previously. When PAO1 is grown in the presence of 1 mM CDCA, we observed the same rate of inhibition of pyocyanin production as in the previous experiment: 38% against 43% (Table 5). On the other hand, the rate of inhibition of pyocyanin production in the presence of 1 mM LCA is only 58%, significantly lower than the rate observed in the two previous experiments (Table 5 and Table 6). However, the inhibitory effect on pyocyanin production was still present.

With the two mutants, the rate of pyocyanin production is of the same order of magnitude as that of the isogenic strain PAO1. On the other hand, the rates of pyocyanin production after exposure of the mutants to BAs were different from those observed with the PAO1 strain. If we consider the results of the exposure of the mutants to CDCA we can observe a difference in behavior between the two mutants. The mutant in the PA1650 gene was sensitive to the presence of CDCA because we observed an inhibition of pyocyanin production of 20%. On the other hand, when the mutant in the PA3264 gene was exposed to CDCA there was no longer any inhibition of pyocyanin production. It therefore seemed that CDCA should enter the bacteria through the Na^+^-dependent transporter product of this gene.

If we now look at the results of the exposure of the mutants to LCA, we can observe a conservation of an inhibitory effect on the production of pyocyanin. The values observed were different from that obtained with the isogenic PAO1 strain: 47% for the mutant in the PA1650 gene and 33% for the mutant in the 3264 gene compared to 58% for PAO1. It therefore seemed that LCA could enter the bacteria using several transporters including the products of the PA1650 and PA3264 genes, with a more important role for the latter.

However, it should be noted that during the comparative analysis of the amino acid sequences of eukaryotic bile acid transporters by BLAST-P against PAO1 proteins two potential homologs of BSEP were identified, whereas there is no homolog in PAO1 for Ostα/Ostβ and a very low score for OATP. The candidates were the PA4997 gene product with 35% amino acids sequence identity and the PA1113 gene product with 33% sequence identity. The PA4997 gene codes for the ATP-binding protein MsbA responsible for the export of lipid A [50] and the PA1113 gene codes for an ATP-binding protein responsible, among other things, for the entry of carbenicillin and dibenzothiophene [51,52]. These two proteins could be potential candidates for LCA entry, MsbA by facilitated diffusion and the PA1113 gene product for the polycyclic structure of LCA. This hypothesis is compatible with the increase in the hydrophobicity of LCA compared to CDCA by the loss of a hydroxyl group.

## 4. Conclusions

In patients with cystic fibrosis and in most cases, the major pathogen remains *P. aeruginosa* despite the occasional presence of other pathogens. *P. aeruginosa* may be exposed to the presence of primary and secondary bile acids and their conjugates in the lungs following gastric reflux or when they end up in the intestine after ingestion of sputum by patients. In the digestive tract, bacteria can be exposed to fairly high concentrations of bile acids.

In our in vitro model, after exposure of *P. aeruginosa* during 24 h to either 1 mM of the primary bile acids (chenodeoxycholic acid (CDCA) and its conjugate glycochenodeoxycholic acid (GCDCA)), or 1 mM secondary bile acids (deoxycholic acid (DCA) and lithocholic acid (LCA) and the conjugate glycodeoxycholic acid (GDCA)), we were able to notice the following: (1) an absence of toxicity with a growth of treated bacteria identical to that of untreated bacteria and even a significant increase in biomass at 24 h in the presence of LCA, (2) a small decrease in the adhesive capacity of treated bacteria on a cell line representative of colon epithelial cells (HT29 cells), (3) a reduction in the overall cytotoxicity of culture supernatants after treatment with bile acids as well as a loss of virulence of these same bacteria on HT29 cells, (4) a decrease in the production of secreted proteases due to a reduction in the expression of the structural genes *las*B and *apr*A, (5) a reduction in the production of pyocyanin after treatment with BAs, with effects that were of the same type as those observed for protease activity, (6) the importance of oxygen availability on the production of proteases and pyocyanin—bacteria growth in Falcon tubes in low LB volume should mimic what happens in a cystic fibrosis patient’s lung—and (7) the possibility that CDCA enters the bacteria via the PA3264 gene product, a homolog of the eukaryotic transporters NTCP1 and NTCP2.

It therefore seemed that *P. aeruginosa* exposed to bile acids lost part of its virulence factors production, following the effects of bile acids on the regulatory elements of quorum sensing as previously observed [22]. Furthermore, in enterocytes and colon epithelial cells, CDCA and secondary bile acids LCA and DCA activate the expression of the gene encoding the farnesoid X receptor (FXR), which modulates the expression of several genes involved in bile acid homeostasis and their transport. The CDCA and secondary bile acids LCA and DCA also control the virulence of bacteria by blocking their invasive capabilities at the level of intestinal villi and cell junctions [53]. Once activated, FXR also induces the expression of SHP (small heterodimer partner), a nuclear receptor that suppresses the synthesis of bile acids, negatively regulates NTCP, and positively regulates BSEP [54,55]. Bile acids also facilitate biofilm formation by *P. aeruginosa* and its tolerance to several antibiotics. They also repress the type III secretion system, the swarming, and the phenazine production [56,57]. It has also been shown that bile in the CF patients’ lungs is a chemoattractant promoting *P. aeruginosa* to adopt a chronic lifestyle [58]. The presence of bile acids associated with reduced oxygen availability in cystic fibrosis patients’ lungs have a direct effect on the production of virulence factors and adhesion capabilities of *P. aeruginosa* and should render it less aggressive, which would allow it to persist in the lungs.

## Figures and Tables

**Table 1:** Adhesion index of *P. aeruginosa* PAO1 on HT29 cells after bacterial growth in presence or absence of bile acids.

Bacteria Treatment	Adhesion Index
None	2.95 ± 0.13
CDCA	2.98 ± 0.23
GCDCA	2.15 ± 0.2
DCA	2.11 ± 0.32
GDCA	2.16 ± 0.11
LCA	2.52 ± 0.13

PAO1 was grown in 25 mL of LB at 37 °C with shaking for 24 h in the absence or presence of 1 mM bile acids. Bacteria were collected by centrifugation and suspended in unsupplemented DMEM, and then 5 × 10^8^ bacteria in 1 mL were added to HT29 cells at 70% confluence on glass cover slips in a 24-well plate during 3 h at 37 °C. After incubation the medium was removed and after washing the cells the adhering bacteria were stained with Geimsa. The cover slips carrying the cells were glued to a microscope slide and the adhering bacteria to 2 × 100 cells were counted. Mean values plus standard deviation (SD) reported in the table were obtained by the average of the values resulting from two independent experiments carried out under the same conditions. The first experiment was carried out in triplicate and the second in duplicate.

**Table 2:** Protease activities in the culture supernatant of PAO1 exposed for 24 h to BAs at 37 °C with shaking in a 250 mL Erlenmeyer flask (volume of LB 25 mL).

Treatment	Specific Protease Activity (mg Azocasein Hydrolyzed/min/mg of Secreted Proteins)	Inhibition (%)
None	0.542 ± 0.082	
CDCA	0.423 ± 0.043	21.98 ± 2.25 (*p* = 0.017)
GCDCA	0.432 ± 0.048	20.26 ± 2.24 (*p* = 0.038)
DCA	0.527 ± 0.082	2.64 ± 0.41 (*p* = 0.014)
GDCA	0.469 ± 0.068	13.33 ± 1.93 (*p* = 0.174)
LCA	0.362 ± 0.045	33.25 ± 4.10 (*p* = 0.003)

After growth, bacteria were centrifuged and the supernatants were collected. The proteins present in the supernatants were concentrated twice in a dialysis hose on a PEG bed. The presence of proteases was detected by measuring the hydrolysis of azocasein. Mean values with SD reported in the table were obtained by the average of the values resulting from three independent experiments carried out under the same conditions. The *p*-values associated with each percentage of inhibition were obtained by the Fisher–Snedecor variance analysis test.

**Table 3:** Protease activities in the culture supernatant of PAO1 exposed for 24 h to BAs at 37 °C with shaking in a 50 mL Falcon tube (LB volume 6 mL).

Treatment	Specific Protease Activity (mg Azocasein Hydrolyzed/min/mg of Secreted Proteins)	Inhibition (%)
None	1.329 ± 0.127	
CDCA	0.809 ± 0.236	39.14 ± 11.42 (*p* = 0.032)
GCDCA	0.328 ± 0.031	75.35 ± 7.23 (*p* = 0.007)
DCA	0.745 ± 0.176	43.99 ± 10.38 (*p* = 0.009)
GDCA	0.489 ± 0.109	63.22 ± 14.10 (*p* = 0.0004)
LCA	0.349 ± 0.113	73.74 ± 23.97 (*p* = 0.019)

After growth, bacteria were centrifuged and the supernatants were collected. The proteins present in the supernatants were concentrated five times by precipitation with ammonium sulfate at 90% saturation. The presence of proteases was detected by measuring the hydrolysis of azocasein. Mean values with SD reported in the table were obtained by the average of the values resulting from three independent experiments carried out under the same conditions. The *p*-values associated with each percentage of inhibition were obtained by the Fisher–Snedecor variance analysis test.

**Table 4:** Inhibition of *las*B, *apr*A, and *nan*A gene expression after treatment of PAO1 with BAs. The mean values with their standard deviations from the mean reported in the table represent the results of the analysis of agarose gels resulting from 2 independent RT-PCRs.

	Percentage of Inhibition of Gene Expression		
Treatment	*las*B	*apr*A	*nan*A
None	0.00	0.00	0.00
DCA	3.2 ± 1.4	5.3 ± 7.1	4.7 ± 5
GDCA	38.7 ± 8.4	15.8 ± 3.2	4.2 ± 2.9
CDCA	28 ± 7	13.5 ± 2.5	2.7 ± 3.6
GCDCA	15 ± 5	20 ± 9	0.00
LCA	37 ± 17	45.7 ± 10.5	0.00

**Table 5:** Pyocyanin production in the culture supernatant of PAO1 exposed for 24 h to BAs at 37 °C with shaking in a 250 mL Erlenmeyer flask (volume of LB 25 mL).

Treatment	Pyocyanine Production (µg/10^9^ Bacteria)	Inhibition (%)
None	3.39 ± 0.26	
CDCA	1.93 ± 0.57	43 ± 0.3 (*p* = 0.25)
GCDCA	1.34 ± 0.19	61 ± 0.9 (*p* = 0.05)
DCA	1.57 ± 0.19	54 ± 0.6 (*p* = 0.17)
GDCA	1.75 ± 0.28	49 ± 0.8 (*p* = 0.12)
LCA	0.91 ± 0.18	73 ± 0.15 (*p* = 0.01)

After centrifugation, the supernatants were filtered through a sterile Millipore 0.45 µm filter to eliminate bacteria that had not sedimented. Then pyocyanin was measured in each supernatant by measuring absorbance at 690 nm, directly or after extraction with chloroform. Results are expressed in µg of pyocyanin produced by 1 × 10^9^ bacteria. The mean values with SD reported in the table were obtained by the average of the values resulting from three independent experiments carried out under the same conditions. The *p*-values associated with each percentage of inhibition were obtained by the Fisher–Snedecor variance analysis test.

**Table 6:** Production of pyocyanin in the culture supernatant of PAO1 exposed for 24 h to BAs at 37 °C with shaking in a 50 mL Falcon tube (LB volume 6 mL).

Treatment	Pyocyanine Production (µg/10^9^ Bacteria)	Inhibition (%)
None	1.54 ± 0.18	
CDCA	1.00 ± 0.09	35 ± 0.3 (*p* = 0.004)
GCDCA	0.85 ± 0.04	45 ± 0.2 (*p* = 0.05)
DCA	1.03 ± 0.06	33 ± 0.2 (*p* = 0.022)
GDCA	1.39 ± 0.12	10 ± 0.1 (*p* = 0.013)
LCA	0.35 ± 0.02	77 ± 0.5 (*p* = 0.04)

After centrifugation, the supernatants were filtered through a sterile Millipore 0.45 µm filter to eliminate bacteria that had not sedimented. Then, pyocyanin was measured in each supernatant by measuring absorbance at 690 nm, directly or after extraction with chloroform. Results are expressed in µg of pyocyanin produced by 1 × 10^9^ bacteria. Values reported in the table were obtained by the average of the values resulting from three independent experiments carried out under the same conditions. The *p*-values associated with each percentage of inhibition were obtained by the Fisher–Snedecor variance analysis test.

**Table 7:** Pyocyanin production by PAO1 and two mutants in potential bile acid transporters after exposure to 1 mM CDCA or LCA.

Strain	Treatment	Pyocyanine Production (µg/10^9^ Bacteria)	Inhibition (%)
PAO1	none	4.18 ± 0.04	0
PAO1	CDCA	2.59 ± 0.06	38 ± 0.6 (*p* = 0.06)
PAO1	LCA	1.74 ± 0.04	58 ± 0.5 (*p* = 0.005)
PW3935 (gene PA1650)	none	4.38 ± 0.35	0
PW3935 (gene PA1650)	CDCA	3.59 ± 0.09	20 ± 0.6 (*p* = 0.05)
PW3935 (gene PA1650)	LCA	2.34 ± 0.06	47 ± 0.8 (*p* = 0.08)
PW6477 (gene PA3264)	none	3.70 ± 0.05	0
PW6477 (gene PA3264)	CDCA	3.72 ± 0.02	0 (*p* = 0.05)
PW6477 (gene PA3264)	LCA	2.46 ± 0.08	33 ± 0.8 (*p* = 0.08)

PAO1, PW3935, and PW6477 were grown in 25 mL of LB medium in the presence of 1 mM CDCA or LCA at 37 °C for 24 h with stirring. Bacterial growth was estimated by measuring turbidity at 600 nm. Bacteria were centrifuged for 5 min at 10,000× *g* and supernatants were collected to measure the absorbance at 690 nm on 1 mL. The pyocyanin concentration is calculated from the absorbance: A690 nm/16 = [pyocyanin] in mg/mL. The quantity of pyocyanin produced by each bacterial strain is expressed in µg per 1 × 10^9^ bacteria. Mean values with SD reported in the table were obtained by the average of the values resulting from three independent experiments carried out under the same conditions. The *p*-values associated with each percentage of inhibition were obtained by the Fisher–Snedecor variance analysis test.

## Data Availability

Data are included in the article.

## References

[1] WHO (World Health Organisation) (2011). Global Status Report on Non Communicable Diseases 2010.

[2] Dickson R.P., Erb-Downward J.R., Freeman C.M., McCloskey L., Falkowski N.R., Huffnagle G.B., Curtis J.L. (2017). Bacterial Topography of the Healthy Human Lower Respiratory Tract. mBio.

[3] Blainey P.C., Milla C.E., Cornfield D.N., Quake S.R. (2012). Quantitative Analysis of the Human Airway Microbial Ecology Reveals a Pervasive Signature for Cystic Fibrosis. Sci. Transl. Med..

[4] Cox M.J., Allgaier M., Taylor B., Baek M.S., Huang Y.J., Daly R.A., Karaoz U., Andersen G.L., Brown R., Fujimura K.E. (2010). Airway Microbiota and Pathogen Abundance in Age-Stratified Cystic Fibrosis Patients. PLoS ONE.

[5] Boutin S., Graeber S.Y., Weitnauer M., Panitz J., Stahl M., Clausznitzer D., Kaderali L., Einarsson G., Tunney M.M., Elborn J.S. (2015). Comparison of Microbiomes from Different Niches of Upper and Lower Airways in Children and Adolescents with Cystic Fibrosis. PLoS ONE.

[6] Nguyen L.D.N., Viscogliosi E., Delhaes L. (2015). The lung mycobiome: An emerging field of the human respiratory microbiome. Front. Microbiol..

[7] Surette M.G. (2014). The Cystic Fibrosis Lung Microbiome. Ann. Am. Thorac. Soc..

[8] Little W., Black C., Smith A. (2021). Clinical Implications of Polymicrobial Synergism Effects on Antimicrobial Susceptibility. Pathogens.

[9] Zolin A., Orenti A., Naehrlich L., Jung A., van Rens J., Fox A., Krasnyk M., Cosgriff R., Hatziagorou E., Mei-Zahav M. (2020). ECFS Patient Registry Annual Data Report 2018.

[10] Guss A.M., Roeselers G., Newton I.L.G., Young C.R., Klepac-Ceraj V., Lory S., Cavanaugh C.M. (2010). Phylogenetic and metabolic diversity of bacteria associated with cystic fibrosis. ISME J..

[11] Chmiel J.F., Davis P.B. (2003). State of the Art. Why do the lungs of patients with cystic fibrosis become infected and why can’t they clear the infection?. Respir. Res..

[12] Muhlebach M.S., Sha W., MacIntosh B., Kelley T.J., Muenzer J. (2019). Metabonomics reveals altered metabolites related to inflammation and energy utilization at recovery of cystic fibrosis lung exacerbation. Metab. Open.

[13] Reen F.J., Flynn S., Woods D.F., Dunphy N., Chróinín M.N., Mullane D., Stick S., Adams C., O’Gara F. (2016). Bile signalling promotes chronic respiratory infections and antibiotic tolerance. Sci. Rep..

[14] Esteller A. (2008). Physiology of bile secretion. World J. Gastroenterol..

[15] Ridlon J.M., Kang D.-J., Hylemon P.B. (2006). Bile salt biotransformations by human intestinal bacteria. J. Lipid Res..

[16] Chiang J.Y. (2009). Bile acids: Regulation of synthesis. J. Lipid Res..

[17] Dawson P. (2001). Role of the intestinal bile acid transporters in bile acid and drug disposition. Handb. Exp. Pharmacol..

[18] Bernstein H., Bernstein C., Payne C.M., Dvorakova K., Garewal H. (2005). Bile acids as carcinogens in human gastrointestinal cancers. Mutat. Res./Rev. Mutat. Res..

[19] Colombo C., Russo M.C., Zazzeron L., Romano G. (2006). Liver disease in cystic fibrosis. J. Pediatr. Gastroenterol. Nutr..

[20] Bernstein C., Bernstein H., Payne C.M., Beard S.E., Schneider J. (1999). Bile salt activation of stress response promoters in *Escherichia coli*. Curr. Microbiol..

[21] Rosenberg E.Y., Bertenthal D., Nilles M.L., Bertrand K.P., Nikaido H. (2003). Bile salts and fatty acids induce the expression of *Escherichia coli* AcrAB multidrug efflux pump through their interaction with Rob regulatory protein. Mol. Microbiol..

[22] Reen F.J., Woods D.F., Mooij M.J., Adams C., O’Gara F. (2012). Respiratory pathogens adopt a chronic lifestyle in response to bile. PLoS ONE.

[23] Laursen J.B., Nielsen J. (2004). Phenazine natural pruducts: Biosynthesis, Synthetic analogues and biological activity. Chem. Rev..

[24] Denning M., Railsback M.A., Rasmussen G.T., Cox C.D., Britigan B.E. (1998). Pseudomonas pyocyanine alters calcium signaling in human airway epithelial cells. Am. J. Physiol..

[25] Lau G.W., Hassett D.J., Ran H., Kong F. (2004). The role of pyocyanin in *Pseudomonas aeruginosa* infection. Trends Mol. Med..

[26] Morihara K., Tsuzuki H., Oka T., Inoue H., Ebata M. (1965). *Pseudomonas aeruginosa* elastase: Isolation, crystallization, and preliminary characterization. J. Biol. Chem..

[27] Heck L.W., Morihara K., McRae W.B., Miller E.J. (1986). Specific cleavage of human type III and IV collagens by *Pseudomonas aeruginosa* elastase. Infect. Immun..

[28] Heck L.W., Morihara K., Abrahamson D.R. (1986). Degradation of soluble laminin and depletion of tissue-associated basement membrane laminin by *Pseudomonas aeruginosa* elastase and alkaline protease. Infect. Immun..

[29] Azghani A.O., Gray L.D., Johnson A.R. (1993). A bacterial protease perturbs the paracellular barrier function of transporting epithelial monolayers in culture. Infect. Immun..

[30] Doring G., Goldstein W., Roll A., Schiotz P.O., Hoiby N., Botzenhart K. (1985). Role of *Pseudomonas aeruginosa* exoenzymes in lung infections of patients with cystic fibrosis. Infect. Immun..

[31] Heck L.W., Alarcon P.G., Kulhavy R.M., Morihara K., Russell M.W., Mestecky J.F. (1990). Degradation of IgA proteins by *Pseudomonas aeruginosa* elastase. J. Immunol..

[32] Braun P., Ockhuijsen C., Eppens E., Koster M., Bitter W., Tommassen J. (2001). Maturation of *Pseudomonas aeruginosa* elastase. Formation of the disulfide bonds. J. Biol. Chem..

[33] Rumbaugh K.P., Griswold J.A., Hamood A.N. (2000). The role of quorum sensing in the in vivo virulence of *Pseudomonas aeruginosa*. Microbes Infect..

[34] Moghaddam M.M., Khodi S., Mirhosseini A. (2014). Quorum sensing in bacteria and a glance on *Pseudomonas aeruginosa* clinical microbiology. Clin. Microbial..

[35] Goldberg J.B., Ohman D.E. (1987). Activation of an elastase precursor by the lasA gene product of *Pseudomonas aeruginosa*. J. Bacteriol..

[36] Goldberg J.B., Ohman D.E. (1987). Cloning and transcriptional regulation of the elastase lasA gene in mucoid and nonmucoid *Pseudomonas aeruginosa*. J. Bacteriol..

[37] Schad P.A., Iglewski B.H. (1988). Nucleotide sequence and expression in *Escherichia coli* of the *Pseudomonas aeruginosa* lasA gene. J. Bacteriol..

[38] Kessler E., Safrin M., Gustin J.K., Ohman D.E. (1998). Elastase and the LasA protease of *Pseudomonas aeruginosa* are secreted with their propeptides. J. Biol. Chem..

[39] Kessler E., Safrin M., Olson J.C., Ohman D.E. (1993). Secreted LasA of *Pseudomonas aeruginosa* is a staphylolytic protease. J. Biol. Chem..

[40] Nouwens A.S., Beatson S.A., Whitchurch C.B., Walsh B.J., Schweizer H.P., Mattick J.S., Cordwell S.J. (2003). Proteome analysis of extracellular proteins regulated by the las and rhl quorum sensing systems in *Pseudomonas aeruginosa* PAO1. Microbiology.

[41] Van Delden C., Iglewski B.H. (1998). Cell-to-cell signaling and *Pseudomonas aeruginosa* infections. Emerg. Infect. Dis..

[42] Gambello M.J., Kaye S., Iglewski B.H. (1993). LasR of *Pseudomonas aeruginosa* is a transcriptional activator of the alkaline protease gene (*apr*) and an enhancer of exotoxin A expression. Infect. Immun..

[43] Antunes L.C., Ferreira R.B., Buckner M.M., Finlay B.B. (2010). Quorum sensing in bacterial virulence. Microbiology.

[44] Caldwell C.C., Chen Y., Goetzmann H.S., Hao Y., Borchers M.T., Hassett D.J., Young L.R., Mavrodi D., Thomashow L., Lau G.W. (2009). *Pseudomonas aeruginosa* exotoxin pyocyanin causes cystic fibrosis airway pathogenesis. Am. J. Pathol..

[45] Heurlier K., Dénervaud V., Pessi G., Reimmann C., Haas D. (2003). Negative Control of Quorum Sensing by RpoN (σ^54^) in *Pseudomonas aeruginosa* PAO1. J. Bact..

[46] Reimmann C., Beyeler M., Latifi A., Winteler H., Foglino M., Lazdunski A., Haas D. (1997). The global activator GacA of *Pseudomonas aeruginosa* PAO positively controls the production of the autoinducer N-butyryl-homoserine lactone and the formation of the virulence factors pyocyanin, cyanide, and lipase. Mol. Microbiol..

[47] Coggan K.A., Wolfgang M.C. (2012). Global Regulatory Pathways and Cross-talk Control *Pseudomonas aeruginosa* Environmental Lifestyle and Virulence Phenotype. Curr. Issues Mol. Biol..

[48] Sabra W., Kim E.-J., Zeng A.-P. (2002). Physiological responses of *Pseudomonas aeruginosa* PAO1 to oxidative stress in controlled microaerobic and aerobic cultures. Microbiology.

[49] Li T., Chiang J.Y.L. (2012). Bile Acid signaling in liver metabolism and diseases. J. Lipids.

[50] Padayatti P.S., Lee S.C., Stanfield R.L., Wen P.-C., Tajkhorshid E., Wilson I.A., Zhang Q. (2019). Structural insights into the Lipid A transport pathway in MsbA. Structure.

[51] Hulen C., Racine P.-J., Chevalier S., Feuilloley M., Lomri N.-E. (2020). Identification of the PA1113 Gene Product as an ABC Transporter Involved in the Uptake of Carbenicillin in *Pseudomonas aeruginosa* PAO1. Antibiotics.

[52] Noda K.-I., Watanabe K., Maruhashi K. (2003). Isolation of the *Pseudomonas aeruginosa* gene affecting uptake of Dibenzothiophene in *n*-tetradecane. J. Biosci. Bioeng..

[53] Inagaki T., Moschetta A., Lee Y.-K., Peng L., Zhao G., Downes M., Yu R.T., Shelton J.M., Richardson J.A., Repa J.J. (2006). Regulation of antibacterial defense in the small intestine by the nuclear bile acid receptor. Proc. Natl. Acad. Sci. USA.

[54] Rizzo G., Renga B., Mencarelli A., Pellicciari R., Fiorucci S. (2005). Role of FXR in regulating bile acid homeostasis and relevance for human diseases. Curr. Drug Targets Immune Endocr. Metab. Disord..

[55] Attinkara R., Mwinyi J., Truninger K., Regula J., Gaj P., Rogler G., Kullack-Ublick G.A., Eloranta J.J., The Swiss IBD Cohort Study Group (2012). Association of genetic variation in the NR1H4 gene, encoding the nuclear bile acid receptor FXR, with inflammatory bowel disease. BMC Res. Notes.

[56] Woods D.F., Flynn S., Caparrós-Martín J.A., Stick S.M., Reen F.J., O’Gara F. (2021). Systems biology and bile acid signalling in microbiome-host interactions in the cystic fibrosis lung. Antibiotics.

[57] Bayat M., Nahand J.S., Farsad-Akhatr N., Memar M.Y. (2023). Bile effects on the *Pseudomonas aeruginosa* pathogenesis in cystic fibrosis patients with gastroesophageal reflux. Heliyon.

[58] Behroozian S., Sampedro I., Dhodary B., Her S., Yu Q., Stanton B.A., Hill J.E. (2022). *Pseudomonas aeruginosa* PAO1 is attracted to Bovine Bile in a novel, Cystic Fibrosis-derived bronchial epithelial cell model. Microorganisms.

